# Optogenetic Delay of Status Epilepticus Onset in an *In Vivo* Rodent Epilepsy Model

**DOI:** 10.1371/journal.pone.0062013

**Published:** 2013-04-24

**Authors:** Inna Sukhotinsky, Alexander M. Chan, Omar J. Ahmed, Vikram R. Rao, Viviana Gradinaru, Charu Ramakrishnan, Karl Deisseroth, Ania K. Majewska, Sydney S. Cash

**Affiliations:** 1 Department of Neurology, Massachusetts General Hospital and Harvard Medical School, Boston, Massachusetts, United States of America; 2 Medical Engineering and Medical Physics, Harvard-MIT Division of Health Science & Technology, Cambridge, Massachusetts, United States of America; 3 Department of Bioengineering, Stanford University, Stanford, California, United States of America; 4 Department of Neurobiology and Anatomy, Center for Visual Science, University of Rochester, Rochester, New York, United States of America; University of California, Riverside, United States of America

## Abstract

Epilepsy is a devastating disease, currently treated with medications, surgery or electrical stimulation. None of these approaches is totally effective and our ability to control seizures remains limited and complicated by frequent side effects. The emerging revolutionary technique of optogenetics enables manipulation of the activity of specific neuronal populations *in vivo* with exquisite spatiotemporal resolution using light. We used optogenetic approaches to test the role of hippocampal excitatory neurons in the lithium-pilocarpine model of acute elicited seizures in awake behaving rats. Hippocampal pyramidal neurons were transduced *in vivo* with a virus carrying an enhanced halorhodopsin (eNpHR), a yellow light activated chloride pump, and acute seizure progression was then monitored behaviorally and electrophysiologically in the presence and absence of illumination delivered via an optical fiber. Inhibition of those neurons with illumination prior to seizure onset significantly delayed electrographic and behavioral initiation of status epilepticus, and altered the dynamics of ictal activity development. These results reveal an essential role of hippocampal excitatory neurons in this model of ictogenesis and illustrate the power of optogenetic approaches for elucidation of seizure mechanisms. This early success in controlling seizures also suggests future therapeutic avenues.

## Introduction

Epilepsy is one of the most common neurological disorders and has severe and devastating manifestations. It is characterized by recurrent seizures and is presently treated by medications, surgery or electrical stimulation. Unfortunately, our ability to control seizures with current therapies remains limited. Medications are the mainstay of treatment, but approximately 30% of patients continue to have seizures despite maximal medical management [1], [2], [3], [4], [5], [6]. Furthermore, current therapies act on multiple targets, affecting normal brain function, and often causing adverse effects. Surgical approaches and electrical stimulation are also not completely effective and carry significant risks.

New therapies that precisely act on specific circuit elements during seizures may be more effective than current options, and would minimize adverse side effects. Dramatic developments in using genetics for optical control of neuronal activity have raised the possibility of an entirely new method for studying and controlling seizures [7], [8]. Placing microbial opsins responsive to light of certain wavelengths under control of specific promoters enables targeting of expression to specific neuronal populations, thus allowing control of selected neural circuits.

In this exploratory study we tested the feasibility of the optogenetic approach in an *in vivo* animal model of epilepsy. We used the lithium-pilocarpine rat model as it has been extensively studied and produces very robust seizures reliably with little variability [9], [10], [11], [12]. We hypothesized that light-activated inhibition can impede development of status epilepticus in awake-behaving rodents and decrease the electrographic or behavioral extent of the seizure. To test this, we transduced glutamatergic hippocampal neurons in adult rats using viral vectors carrying a potent optogenetic inhibitor, enhanced halorhodopsin (eNpHR3.0), and fluorescent marker yellow fluorescent protein (YFP) under control of the excitatory neuron-specific calcium/calmodulin-dependent protein kinase II alpha promoter (CaMKIIα) [13], [14], [15], [16]. We examined whether inhibition of eNpHR-expressing excitatory neurons by illumination during seizure onset can modulate the spatial and/or temporal extent of seizure activity and its dynamics. Our results demonstrate the potential for optogenetic approaches for seizure control and the dissection of their complex neural mechanisms. These proof-of-principle experiments are a first step towards developing future optogenetic treatments whereby precise spatiotemporal control over specific circuit elements will target treatment to only those neuronal elements responsible for seizure activity and thereby sparing normal brain function, thus overcoming one of the major disadvantages of current pharmacological treatments.

## Methods

All procedures were performed in accordance with institutional and national guidelines for animal care and use for research purposes and the study protocol was approved by the Massachusetts General Hospital institutional review board (IACUC, protocol # 2009N000051).

### Virus

Recombinant adeno-associated virus (AAV) vector carrying Halorhodopsin (eNpHR) gene tagged with a fluorescent marker protein (yellow fluorescent protein, YFP) under the control of the CaMKIIα promotor was prepared at the University of North Carolina Vector Core Facility (rAAV5/CaMKIIα-eNpHR3.0-eYFP). The titer of the concentrated virus for *in vivo* injection was 3×10^12^ virus molecules/ml.

### Injection/light Delivery System Design

In order to deliver the virus and to manipulate transduced neurons by light while recording neuronal activity we developed a custom designed injection-recording system. It consisted of a pedestal, electrodes and optical fiber guide, through which the viral vector and optical fiber were introduced during the experiment. A custom made plastic pedestal with 6 openings (Plastics One) was secured to the skull using dental acrylic cement (GlassLute, Pulpdent). Five openings were used to connect the reference, two cortical and two hippocampal electrodes. A 20 ga guide cannula was molded into the sixth opening to act as an optical fiber guide. The tip of the twisted electrode used to record from the hippocampus was aimed at the location of the injection. To inject the virus, a 26 ga injection cannula was lowered through the guide cannula into the hippocampus and the virus was deposited. During the experiment, an optical fiber (crossection 200 µm, BFL37–200, ThorLabs) was lowered through this same guide cannula to the stereotaxic position of the virus injection to allow illumination of transduced neurons.

### Recording System

Electrodes were connected to a multi-channel USB Biosignal amplifier (Guger Technologies) via the pedestal using a five channel custom cable (Plastics One) secured to the optical fiber. The optical fiber was coupled to a 561 nm laser (CrystaLaser) through an FC/PC connector. The laser was gated by a digital computer pulse (TTL) delivered via the parallel port of a laptop computer. EEG was recorded at a sampling rate of 256 or 512 Hz with bandpass filtering from 0.1 to 100 Hz. Video recordings were also taken of each experiment for behavioral analysis.

### Surgery: Injection System/electrode Implantation

Adult male Sprague-Dawley rats were used in this study. Rats were anaesthetized with pentobarbital (50 mg/kg). The top of the head was shaved, and the animal was immobilized in a stereotaxic frame. The skin was opened to expose the skull. Three small burr holes were drilled in the skull: two 1 mm anterior to bregma, 1.5 mm lateral to the midline over each hemisphere, and one 4 mm posterior to bregma, 2.5 mm lateral to the midline on the right side. Surface recording electrodes (two stainless steel jeweler’s screws, shaft diameter 1.2 mm, length 1.6 mm, Plastics One) were inserted in the right and left frontal locations. A twisted–wire electrode coupled to the guide cannula was lowered into the hippocampus to a depth of 3 mm according to the atlas of Paxinos and Watson via the third hole [17]. The electrodes were externalized through the pedestal and the installation was mounted to the skull using acrylic cement. For pyramidal neurons transduction, 4–6 µl of viral construct were slowly pressure injected into hippocampus at a rate of 0.5–1 µl/min using a 26 ga injection needle lowered through the guide cannula. The injection needle was left in place for 5 min and then gradually withdrawn. The pedestal was covered with a cap to prevent the clogging of connections. Postoperatively, animals were monitored until awaking and then returned to the animal facility.

### Seizure Induction and Monitoring

Following the recovery period, seizures were induced using the lithium-pilocarpine model. Animals received 127 mg/kg lithium hydrochloride i.p. 18–24 hours prior to injection of pilocarpine for seizure induction. On the experimental day scopolamine methyl bromide 1 mg/kg was injected i.p. 30 min before injection of pilocarpine to prevent peripheral cholinergic-agonist induced side effects. Electrodes were connected through the head pedestal to an EEG amplifier system, and the recording was started. To initiate seizures pilocarpine, a muscarinic cholinergic agonist, (30 mg/kg) was injected i.p. and the recording continued. After seizures developed the recording continued for another 20–30 min and then pentobarbital (100 mg/kg i.p.) was used to stop the seizure. Animals were then perfused transcardially with phosphate buffered saline (PBS) followed by 10% formalin, and the brains were dissected and processed for histology.

### Data Analysis

EEG recordings were downsampled, when necessary, to a common sampling rate of 512 Hz to allow for standardized analyses to be performed across recordings. To obtain time-frequency representations of the seizures, a short-time fast Fourier transform was performed using a sliding Hamming window 1000 ms long, overlapped by 500 ms. Line-length [18] was also computed in sliding 3 second windows (overlapped by 1.5 s) for the entire recording in each animal. The line-length feature reflects the changes in the waveform and is a measure of the amplitude and frequency dynamics of the seizure. To quantify the relationship between the activity in hippocampal and cortical electrodes, phase synchrony and cross-correlation were computed between pairs of electrodes in 1 second windows (overlapped by 500 ms). The frequencies used were 2–50 Hz. All data are presented as mean±SEM. Comparisons between the experimental groups were made using one-way ANOVA, and corrected for multiple comparisons using the Holm-Sidak method.

To characterize the dynamics and evolution of the seizures, cumulative line-length was computed for 25 minutes from the start of each seizure. The cumulative line length, *LL_cum_*, is the running sum of distances between successive points within an expanding window of size n, always starting at point 1, and was defined as
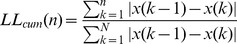
where x[k] is the signal at the k^th^ sample, and N is the total number of points in the signal. This makes the cumulative line length monotonically increasing from 0 to 1 over the length of the signal. A seizure with constant power throughout the 25 minutes would yield a straight line from 0 to 1, a seizure which slowly increased in amplitude would be concave-up and lie below this line, while a seizure which started strong and leveled off would be concave-down and lie above this line. To test for differences between the cumulative line-lengths of the experimental groups, a non-parametric, cluster-based statistical test was utilized [19].

Behavioral seizure manifestations were rated on the Racine scale in 30 second epochs based on review of videorecordings of the experiments [20]. Differences between the group scores were tested using the same non-parametric, cluster-based statistical test as above [19].

### High-gamma Power Analysis

Spectral analysis of the hippocampal LFP was performed using the Chronux toolbox for Matlab [21]. The high-gamma band was defined as 70–110 Hz. Power in the high-gamma power band was calculated over 5 second intervals using a time-bandwidth product of 1 and 1 taper. The high-gamma power in the 5 second period immediately preceding the onset of the light was defined as the baseline power. Power in all subsequent 5 second periods was normalized by dividing by the baseline power. This made it easier to make comparisons across different animals and conditions. Normalized power in subsequent bins was compared to baseline using the Mann-Whitney U test.

### Experimental Design

#### Sham control group

Animals in this group were implanted with electrodes and guide cannula (for optical fiber introduction), but no virus was injected. Animals were allowed to recover for 9–14, 16–21, or 31–35 days after electrode implantation (n = 4, n = 5, n = 4 respectively). Following the recovery period, seizures were induced using the lithium-pilocarpine model.

#### NpHR control group

This group is designed to control for the possible effect of the viral construct *per se*. Seven animals were implanted with electrodes and guide cannula (for optical fiber introduction), and viral construct was injected. Following the recovery period (13–27 days), seizures were induced using the lithium-pilocarpine model, but no light was delivered via the introduced optical fiber.

#### Light control group

This group is designed to control for the possible effect of light. Eight animals were implanted with electrodes and guide cannula (for optical fiber introduction), but no virus was injected. Following the recovery period (10–29 days), seizures were induced using the lithium-pilocarpine model, and light delivered via the optical fiber similar to the experimental group.

#### Experimental groups

Experimental attempts to control seizures by light were conducted 13–23 days following electrode implantation and virus injection. Electrodes and the optical fiber were connected to the recording system/laser as discussed above. The laser output power was set to 35 mW (fiber output power 18 mW as measured using a power meter (PM100D, ThorLabs)), corresponding approximately to light intensity of 570 mW/mm^2^. We based light manipulation parameters on those found in literature in other *in vivo* systems [14],[15]. After 10 minutes of baseline recording, 30 mg/kg pilocarpine was injected i.p. to initiate the seizure. Experiments were conducted using one of the following two protocols.

#### Continuous illumination protocol

Five to ten minutes after injecting pilocarpine (8±0.9 min), irrespective of visualized electrographic activity but designed to be 1–2 min before earliest anticipated seizure start according to our control group data, the laser was continuously activated until the seizure was terminated with pentobarbital overdose.

#### Intermittent/triggered illumination protocol

In a different set of experiments illumination was initiated when a change in the EEG suggestive of early evolving seizure activity or a change in the behavior of the animal was observed. In this circumstance the laser was turned on for periods of 1 to 2 min, with at least 1 minute between light pulses, until the seizure was stopped with pentobarbital overdose. This non-continuous triggered illumination began approximately 7–8 min after injecting pilocarpine (7.4±0.2 min).

Animals from experimental and control groups were alternated. Final histological confirmation of injection and eNpHR expression success was done post-experimentally, therefore investigator was blinded to the experimental outcome.

### Estimation of Tissue Affected by Light

According to Gradinaru et al. (2009), light power sufficient to activate eNpHR (1 mW/mm^2^) is present at least within 1.5 mm distance of the fiber tip using 30 mW light source. We used a laser output power of 35 mW, with measured fiber power output of 18 mW with a 200 µm diameter fiber, corresponding approximately to a light intensity of 570 mW/mm^2^. Based on the light penetration curve for 561 nm light [15], light intensity sufficient to activate eNpHR was present at a distance of at least 2 mm from the light source.

## Results

We attempted to control acute seizures in awake-behaving rats in the lithium-pilocarpine model of elicited seizures using an optogenetic approach. Hippocampal pyramidal neurons were transduced with a virus carrying eNpHR *in vivo* and seizure manipulation attempts were made using light delivered to hippocampus via an optical fiber. Yellow light illumination of transduced neurons prior to seizure onset significantly delayed electrographic and behavioral initiation of status epilepticus, and altered the dynamics of ictal activity development.

### Implant Tolerability and Injection Assessment

The tolerability of the implant used for recordings, virus injection and light delivery to the hippocampus was assessed in a control group of animals. Following the implantation, tissue damage was limited to the immediate proximity of the electrodes (200–400 µm) with no regions devoid of neurons present elsewhere in the hippocampus and cortex at any of the survival time points (12–35 days; n = 12), showing that the implant was well tolerated. In addition, in virally-transduced animals, pyramidal neurons located in the area surrounding the implant expressed eNpHR, suggesting that these neurons were healthy as evidenced by intact cellular machinery.

**Figure 1:**
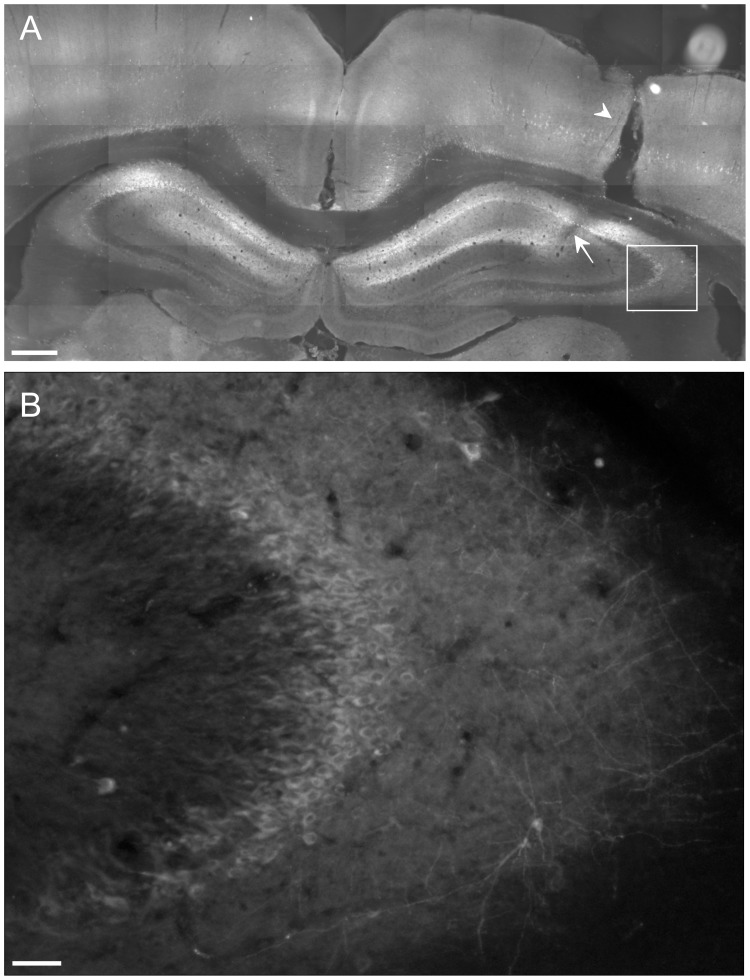
Hippocampal pyramidal neurons expressing eNpHR-EYFP, as visualized by marker protein YFP fluorescence. **A**. Photomicrograph showing bilateral extent of the labeling in hippocampus. **B**. Higher magnification of the boxed region in **A** demonstrating the morphology of transduced neurons in more detail. Arrowhead and arrow designate the tracks left by the guide cannula and recording electrode, respectively. Notice the high density of transduced cells in the hippocampal pyramidal cell layer. Scale bars: 500 µm (A); 50 µm (B).

In animals transduced with AAV, the transduction extent was verified in histological sections of the brain following the experiment. Hippocampal neurons were transduced successfully and virus was expressed predominantly in pyramidal cells and granule cells in dentate gyrus, as demonstrated by fluorescent signal from YFP. The full medio-lateral extent of hippocampal pyramidal layer neurons was labeled (CA1, CA2), with labeling extending to CA3 in the dorso-ventral dimension. Transduced neurons extended over 3.5 mm antero-posteriorly, with minimal spread of at least 2 mm (Fig. 1). Some cortical neuron labeling occurred as well.

### Controls

**Figure 2:**
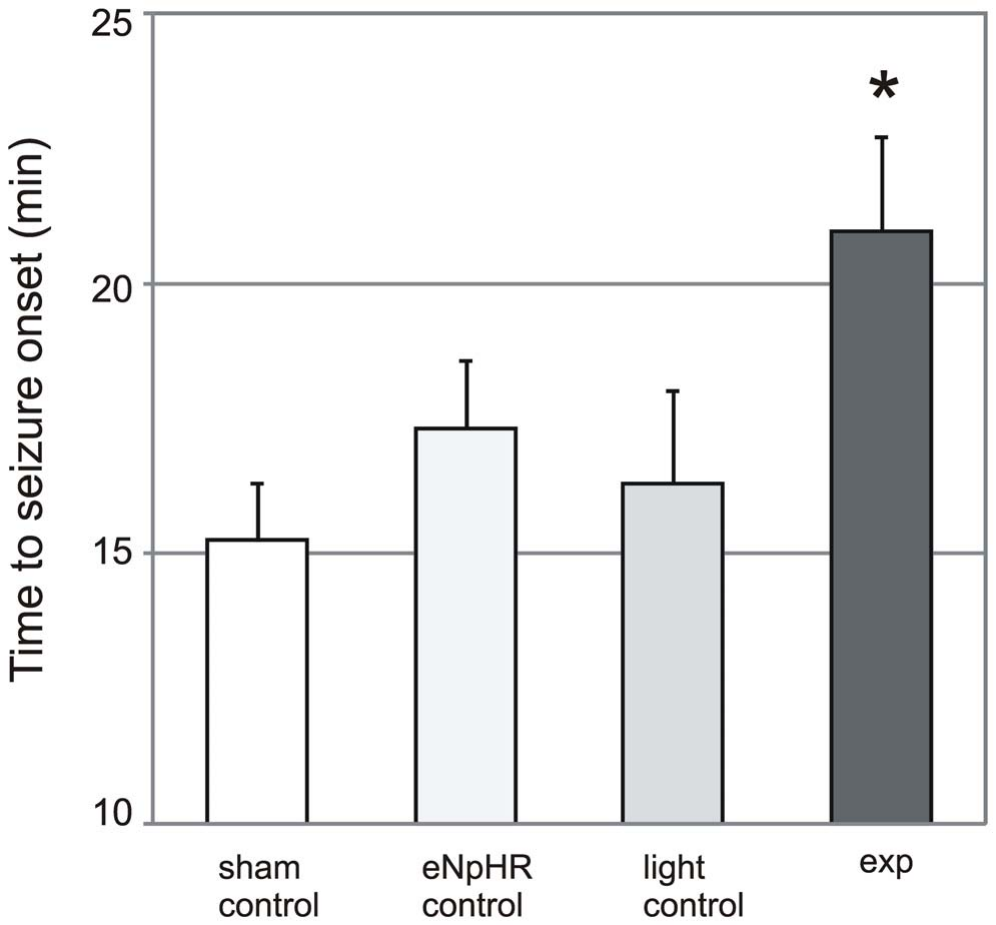
Bar graph showing time to seizure onset following pilocarpine injection in control animals, animals expressing eNpHR with no light manipulation (control eNpHR), light control animals and experimental eNpHR-transfected animals. Experimental group is significantly different from the control group, (p = 0.007, one way ANOVA followed by Holm-Sidak method for multiple comparisons versus the control group). Error bars represent SEMs.

**Figure 3:**
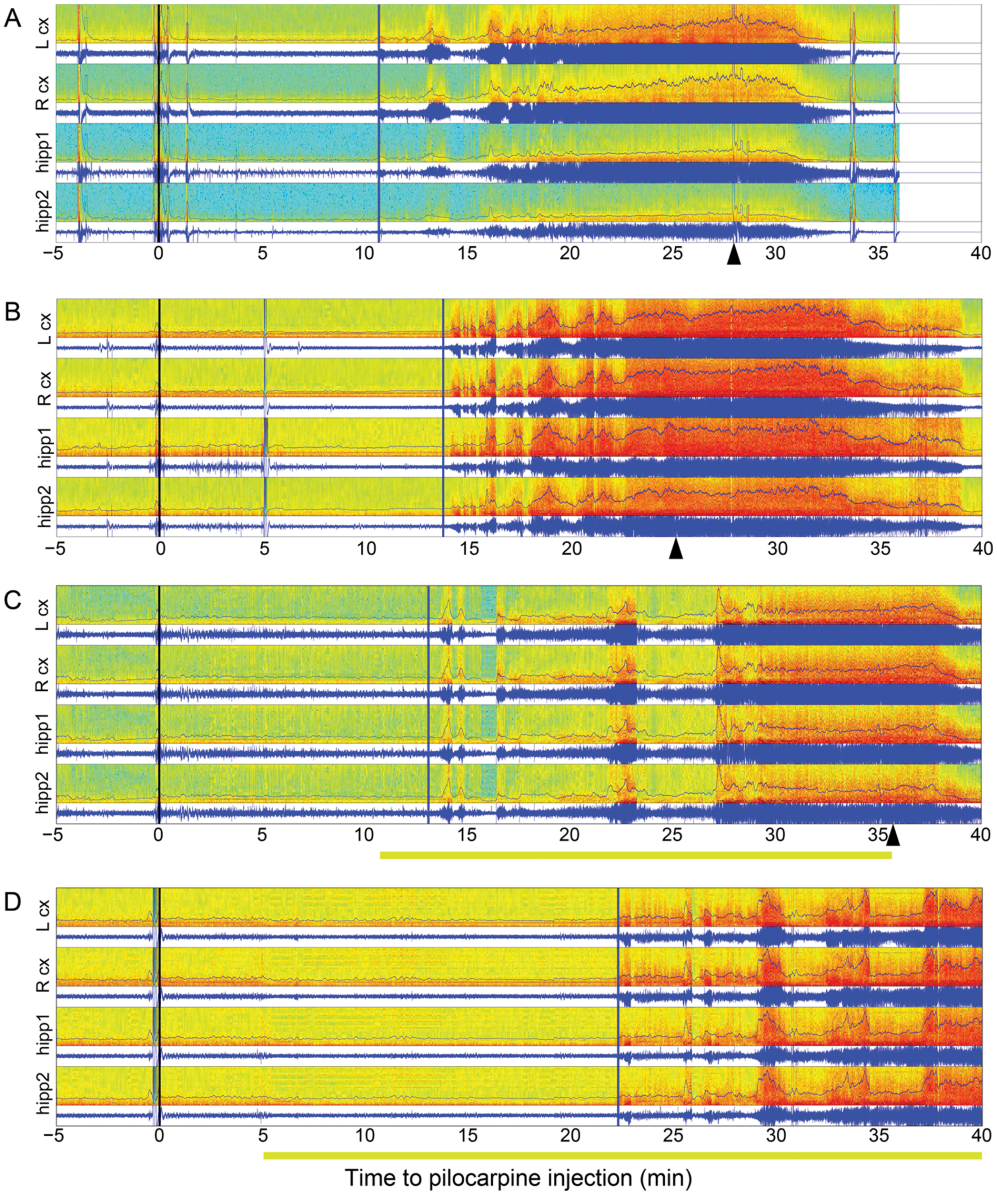
Plot showing EEG recordings in representative examples of sham, transduced non-illuminated and light control animals (A, B and C respectively), and transduced illuminated (D) rats. The seizure was terminated by pentobarbital administration in each case (arrowhead). Color spectrogram represents time-frequency distribution (scale is 0 to 50 Hz) and blue line represents the EEG recording (µV, scale 0 to 600 µV). Black vertical lines mark pilocarpine administration at time 0, vertical blue lines mark the beginning of the seizure. Yellow shading under the time axis indicates illumination with yellow light. Blue traces overlaid on the spectrograms show windowed line-length over the course of the seizure.

**Figure 4:**
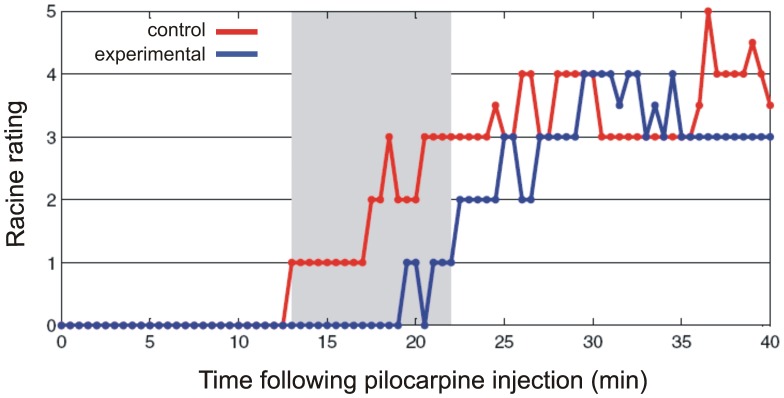
Behavioral seizure progression on the Racine scale in the control (sham, eNpHR, light controls, total of 11 control experiment video recordings were rated) and experimental (N = 17) groups. Behavioral manifestations of the seizure were delayed in experimental animals as compared to the controls. Area shaded in gray marks the timepoints at which group scores differed significantly (non-parametric, cluster-based statistical test, p<0.05).

We first characterized the course of seizure development in a sub-group of sham control animals which were not transduced with eNpHR. After gathering this initial information we conducted experiments with experimental and the different control groups interleaved. Pilocarpine injection reliably induced seizures in all of the control rats. Seizure onsets occurred 15.2±1.1 min following pilocarpine injection (n = 13, Fig. 2, 3). The seizure start was characterized by an almost simultaneous appearance of low frequency, low amplitude epileptiform spike activity on cortical electrodes in both hemispheres and hippocampal electrodes. In most cases this activity was intermittent initially and involved lower frequencies (20 Hz and below) and amplitudes (<500 µV). Over 0.5–3 minutes the activity evolved into continuous spike and spike-wave activity, and subsequently became a fully developed seizure characterized by the presence of higher frequencies (up to 50 Hz) and amplitudes (up to or greater than 500 µV) as has been detailed previously [11]. Immunohistochemical staining demonstrated significant upregulation of activity-regulated immediate early gene Arc expression in hippocampus and especially in dentate gyrus bilaterally following seizure as compared to naïve controls (not shown), as indicative of neuronal activity induced by seizures [22],[23]. Behaviorally, the animals followed a pattern well described for this model, characterized by facial movements (e.g. chewing, jaw-opening), head nodding, automatisms of the extremities, followed by myoclonic jerks and clonic seizures with rearing, falling and loss of postural control [11], [12], [24]. Mild behavioral manifestations (i.e. mouth movements, chewing) preceded the appearance of spike activity on the EEG by 1–3 min (Fig. 4). The evolution of behavioral seizure manifestations accompanied their electrophysiological development.

Two additional control groups were designed to assess for possible effects of the NpHR transgene and the light. In the first, hippocampal neurons were transduced with the virus, the optical fiber was introduced, but no light manipulation was applied during the course of the experiment (NpHR control). The delay before seizure start in this group was similar to that of the original control group with no virus injected (17.3±1.3 min, n = 7, p = 0.4, one way ANOVA followed by Holm-Sidak method for multiple comparisons versus the control group). In the additional control group (light control) no virus was injected and light was delivered via the optical fiber similar to the experimental group. Seizure onset delay in this group was 16.3±1.7 (n = 8) similar to the sham control group (p = 0.66, one way ANOVA followed by Holm-Sidak method for multiple comparisons versus the control group).

To confirm inhibition of transduced hippocampal neurons by light and to control for the effect of prolonged inhibition of pyramidal hippocampal neurons and possible rebound activity after light discontinuation, illumination was tested in animals expressing eNpHR in the absence of seizures. Our experimental design did not allow recording from single units to confirm inhibition directly, therefore we used high frequency oscillations as a surrogate for spiking activity. In particular, high-gamma oscillations in the 70–110 Hz range are strongly correlated to spiking activity [25]. In an animal expressing eNpHR in the absence of seizures, we delivered 5 second pulses of light, separated by intervals of 30 seconds (N = 35 pulses) and analyzed the spectral content of the LFP recorded from the hippocampus (Figure S1A). We found that the averaged high-gamma power was visibly reduced when the light was switched on, in keeping with a reduction of hippocampal activity. In 8 animals expressing eNpHR, light was turned on for one to three minutes and then switched off. In 7 out of 8 animals high-gamma power was significantly reduced during the first 5 seconds of illumination, when compared to baseline levels (Figure S1B, D, p<0.006, Mann-Whitney U test). In subsequent 5 second illumination window high-gamma power was reduced, though not significantly (Figure S1B), and there was no significant reduction in following 5 second windows (data not shown). No rebound electrophysiological activity was observed after illumination.

### Optogenetic Seizure Control *in vivo*

We used two protocols for light-mediated inhibition of seizures: a continuous illumination and an intermittent illumination in which light was turned on for 1–2 minutes at 1 to 3 min intervals. In both protocols the illumination was started 5–10 minutes following pilocarpine injection, a few minutes before the earliest time of the anticipated seizure start according to our control group data. Both modes of illumination delayed the electrographic and behavioral onset of the seizure and changed the dynamic of seizure evolution in the same way. In these light illumination experiments, electrographic seizures started 21±1.8 min following administration of pilocarpine (n = 16, Fig. 2, 3), resulting in an average delay of 6 minutes relative to the sham control group (p = 0.007).

In addition to affecting the time to onset of electrographic and behavioral (Fig. 4) seizure activity, optogentic manipulation also resulted in changes in early seizure dynamics. After the initiation of seizure activity, optogenetic inhibition generally delayed the development of high amplitude continuous oscillations (fully developed seizure) involving 20–50 Hz activity. The seizures of the experimental animals were characterized by an initial period of low-amplitude, low-frequency oscillations with episodes of remission exhibiting almost normal EEG activity. This is in contrast to the sustained and early transition to high frequency, high amplitude activity seen in control animals. In three animals only short bursts of activity occurred around average seizure onset time, followed by normal EEG activity, with clear seizure activity not beginning for another 7–10 min in two animals, and not occurring at all in the third. In this animal light was turned off after 35 min, and seizure activity occurred 15 min later.

**Figure 5:**
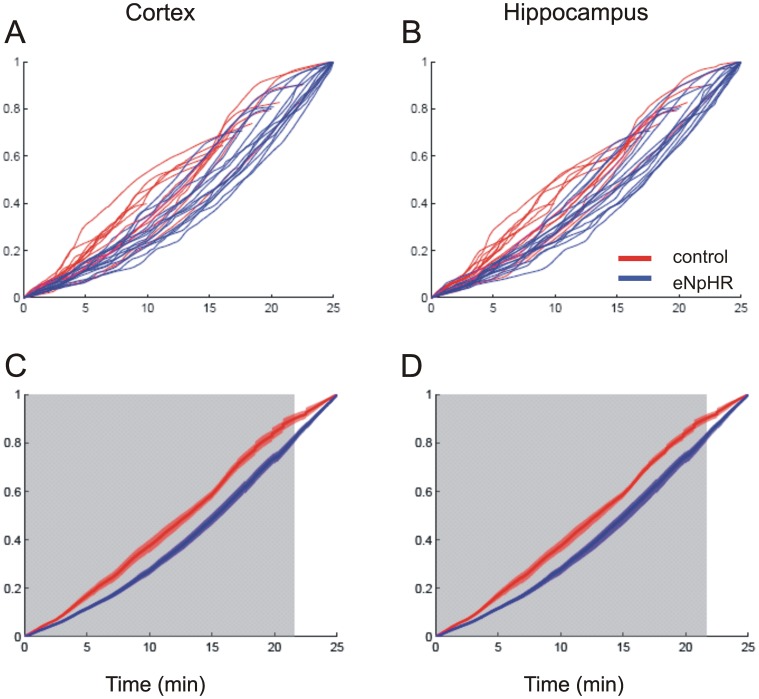
Early seizure dynamics differ with optogenetic manipulation. Cumulative line-length, or path-length, of the cortical EEG recording in control (red, N = 14, from sham and additional control groups) and experimental (blue, N = 16) rats from onset to 25 minutes into the seizure, reflect changes in power and frequency on the standardized scale (Y axis). Areas with shallow slopes indicate lower seizure activity, while steep slopes indicate increased amounts of seizure activity as measured by line-length. **A** and **B** show individual animal plots for cortex and hippocampus, respectively. **C** and **D** show mean path-length ± SEM (represented by line and shading) of the control and experimental animals in cortex and hippocampus, respectively. The gray shaded box designates the time points at which cumulative line-length was statistically different between control and experimental animals (non-parametric, cluster-based statistical test, p<0.05).

To quantify the dynamics of seizure development we calculated the cumulative line-length for cortical and hippocampal channels as a measure of the amplitude and frequency dynamics of each seizure [26]. Experimental animals, compared to controls, showed shallower slopes of cumulative line-length reflecting a slower evolution of seizure activity from initiation to peak seizure activity (Fig. 5; p<0.05). Phase synchrony and cross-correlation between pairs of electrodes (cortical left *vs* right, cortical *vs* hippocampal, hippocampal *vs* hippocampal) decreased following onset of the seizure, especially between cortical and hippocampal electrodes (not shown). There were no consistent changes associated with illumination alone in phase-synchrony or cross-correlation between pairs of the electrodes.

Interestingly, analysis of the spectral content of the LFP recorded from the hippocampus in experimental animals (expressing eNpHR, when light was turned on following seizure induction by pilocarpine) revealed that even in the presence of pilocarpine exerting its proconvulsive effects, 10 out of 12 animals showed significantly reduced high-gamma power during the first 5 seconds of illumination (p<0.004, Mann-Whitney U test; Figure S1C, D), while control animals (not expressing eNpHR) injected with pilocarpine showed no change (n = 7, Figure S1D), further supporting the suggestion that the delay in seizure onset is specifically related to light-mediated inhibition of hippocampal pyramidal cells.

## Discussion

We successfully introduced optogenetic probes into pyramidal neurons of the hippocampus and subsequently recorded from and directed light into those structures in a freely behaving animal. Inhibiting activity in primary excitatory hippocampal neurons with light delayed status epilepticus onset in the lithium-pilocarpine epilepsy model. This result suggests that seizure activity originating in the hippocampus may be crucial for seizure development and subsequent propagation to the cerebral cortex in this model [27], [28]. Our results suggest that inhibiting specific neuronal populations at the seizure initiation zone with light has a potential for controlling epileptic activity.

High-gamma power reflects neuronal spiking activity and its suppression following illumination suggests inhibition of transduced hippocampal neurons by light, as has been demonstrated directly by electrophysiological *in vitro* studies of NpHR-transduced neurons [8], [13]. These data support the hypothesis that the delay in our *in vivo* epilepsy model is due to the inhibitory effect of light on activity of transduced neurons.

The NpHR light-driven inhibition acts by shifting the membrane potential and hyperpolarizing neurons; Zhang et al [13] demonstrated that NpHR mediated inhibition remains stable over long timescales. While intracellular accumulation of chloride in pyramidal neurons could cause a depolarizing shift in the GABA_A_ receptor reversal potential [29], Kokaia’s group [8], [30] showed that activation of transgene NpHR does not alter the reversal potential for the GABA_A_ receptor-mediated monosynaptic inhibitory postsynaptic currents in principal hippocampal neurons in an in vitro epilepsy model. Furthermore, it is unlikely that GABA had become transiently excitatory and contributed to the epileptiform activity in our experiment, because seizure onset was delayed in our experimental design, and gamma power analysis supported silencing of hippocampal pyramidal neurons by NpHR activation.

The fact that unilateral inactivation was sufficient to produce this result raises the possibility that each hippocampus plays a role in the network of activated structures necessary for seizure evolution. Removing one portion of this network alters the ictogenic process. These results are in line with findings reported for kainate model of epilepsy in mice [31], where spatially limited unilateral silencing of pyramidal neurons abolished over half of the seizures.

Two illumination protocols, tested for efficiency and safety reasons, produced similar results, suggesting that the inhibition produced in both modes is sufficient to delay the seizure onset, likely by delaying development of activity to the level necessary to evolve into seizure, while not causing obvious adverse behavioral or electroencephalographic effects. Slower dynamics of early seizure evolvement in experimental animals as demonstrated by cumulative line-length also points toward suppression of neuronal activity and delay in its development leading to interruption in “orchestration” of activity between neuronal populations into high amplitude oscillations. Controls further confirm that seizure delay is due to light-mediated inhibition of the hippocampal neurons expressing eNpHR. First, eNpHR expression by itself does not affect seizure development time, as animals which expressed eNpHR, but were not exposed to light, showed no delay of seizure onset. Second, illumination of transduced hippocampal neurons in the absence of seizure resulted in no rebound activity after light discontinuation. These finding are in line with Tonnesen et al [8], who demonstrated *in vitro* that expression of NpHR transgene alone has relatively limited effects on the properties of neurons. Therefore, the inhibition and subsequent release from inhibition in the intermittent illumination case was unlikely to be adding drive toward ictal activity. Illumination in non-transduced animals did not delay seizure onset and was not associated with decrease in high gamma-power, further proving that the effect was mediated by light manipulation of the transduced neurons.

The modest scale of seizure attenuation in this study is in accordance with the extent of the hippocampal area affected by light and experimental design used. This may have contributed to the fact that illumination did not produce a visible change in cross-correlation or phase synchrony between the electrodes. The study was designed as an exploratory study of applying optogenetic approach for control of seizures, therefore we applied a minimal intervention approach and affected circuitry involved in seizures unilaterally. Even unilateral intervention was powerful enough to produce seizure delay. Since the epilepsy model used in our study is not focal, many of the neurons that contribute to the seizure are beyond the range of the unilateral optical fiber light inhibition. In addition, in this model pilocarpine used to induce the seizure remains present in the system and continues to exert proconvulsant activity long after the start of the light triggered inhibition [32], [33]. In line with our data, Bittencourt and colleagues were able to delay, but not abolish pilocarpine-induced seizures by muscimol microinjection prior to pilocarpine administration [34]. Given this, one would not expect seizures to be completely blocked by our current approach. Future experiments will be necessary to test whether more extensive pyramidal neuron inhibition (e.g. by bilateral or multiple optical fiber arrays) will be required for seizure abrogation by light in this model and to determine its exact extent. In line with this, recent studies have demonstrated possibility of seizure control in focal epilepsy models, where location of the epileptic focus is known and can be specifically targeted [35], [36].

A number of other limitations could be addressed by future experiments. Epilepsy is thought to result from an imbalance in excitatory and inhibitory activity [37], . In this study we limit our approach to inhibiting excitatory neurons. NpHR-YFP was expressed predominantly in pyramidal cells and granule cells in dentate gyrus, as confirmed by morphology, but this was not assessed quantitatively. Possible effects of non-specific expression, such as silencing of inhibitory cells, which could cause pro-epileptogenic activity can’t be completely ruled out, though selectivity of this construct expression under CaMKIIα promotor to excitatory glutamatergic neurons has been demonstrated [8], [15]. An alternative approach may test potentiation of hippocampal inhibitory circuitry, by using viral constructs carrying excitatory channels such as channelrhodopsin under inhibitory neuron promoters. Combining expression of both types of optogenetic modulation constructs may provide the best mode of control, and remains to be tested.

Further steps will include optimizing the timing of light manipulation with regard to prospective seizure onset [40]. There is a growing evidence for ictal activity present in small domains [41], [42] or at the level of individual neurons [43], before the onset of a seizure is visible on EEG or has a behavioral manifestation. These periods, when ictal activity is most spatially confined might be the most susceptible to optogenetic control. An automatic light triggering system responding to local electrographic activity will enable more precise closed control loop.

In summary, our results demonstrate that inhibition of excitatory drive in hippocampus can delay seizure onset. This provides a proof-of-principle that this approach could be used in the future for treatment of seizures and the dissection of epilepsy mechanisms.

## Supporting Information

Figure S1 **High-gamma power is transiently suppressed during illumination.** **A.** Averaged LFP spectrogram in response to 5 second pulses of light delivered with 30 second intervals between each pulse (N = 35 pulses). Note the clear decrease in high-gamma power across most frequencies during the periods of illumination (green bar). **B, C.** High-gamma power is decreased during the first 5 seconds of prolonged illumination (lasting 1–3 minutes) in animals expressing eNpHR, but not during any subsequent 5 second periods. This is true both in the absence (**B**) and presence (**C**) of pilocarpine to induce seizures. **D.** Bar and whisker plot of changes in high-gamma power during the first 5 seconds of illumination. Power is decreased in animals expressing eNpHR (red and blue bars), but is non-significantly increased in control animals that do not express eNpHR. This suggests that light-activated hyperpolarization of eNpHR expressing pyramidal cells is playing a role in decreasing high-gamma power. Median (horizontal line), 25–75 percentiles (box) and 2.5–97.5 percentile of the data (whiskers) are shown.
(TIFF)

Methods S1 **Histological processing of the brain and immunohistochemistry.** The brains were dissected and processed for histology for injection site/electrode location and virus expression confirmation. Following postfixation in 10% formalin for 2–4 hours at room temperature, brains were cryoprotected in PBS containing 20% sucrose and 0.02% sodium azide at 4°C, cut in serial coronal sections 40 µm thick on a freezing microtome and stored in PBS-azide. Sections were mounted on slides and evaluated for direct identification of YFP expressing cells using fluorescent microscope. For histological evaluation sections were stained with Cresyl Violet or Hematoxylin and Eosin. Fluorescence immunostaining of free-floating sections was performed to visualize endogenous expression of activity-regulated immediate early gene Arc and to amplify YFP signal from the viral transgene. Briefly, sections were blocked for 1 hour at room temperature with 10% (v/v) normal goat serum (NGS, Jackson Immunoresearch) in PBS containing 0.1% (v/v) Triton X-100 (PBST) and then incubated overnight at 4°C with primary antibodies diluted in PBST containing 3% NGS. A monoclonal mouse anti-GFP antibody that recognizes YFP (clone 3E6, Invitrogen) was used at 1∶1000 dilution, and a polyclonal rabbit anti-Arc antibody (gift of Dr. P. Worley) was used at 1∶4000 dilution. After four rinses with PBST, sections were incubated for 2 hours at room temperature with secondary antibodies, Alexa 488-conjugated goat anti-mouse IgG and Alexa 633-conjugated goat anti-rabbit IgG (Invitrogen), each at 1∶250 dilution. Sections were rinsed four times with PBST and mounted on slides with Fluoromount (Sigma-Aldrich). Sections were evaluated and images acquired using an inverted epifluorescence microscope (Nikon) equipped with appropriate filters to selectively visualize each fluorophore, and montages covering the hippocampus were assembled using Volocity software (PerkinElmer).
(DOCX)
