# Supplementary figures and images for: Optogenetic Delay of Status Epilepticus Onset in an In Vivo Rodent Epilepsy Model
Source: PLoS One. 2013 Apr 24;8(4):e62013. doi: 10.1371/journal.pone.0062013 (PMC3634849; doi:10.1371/journal.pone.0062013)

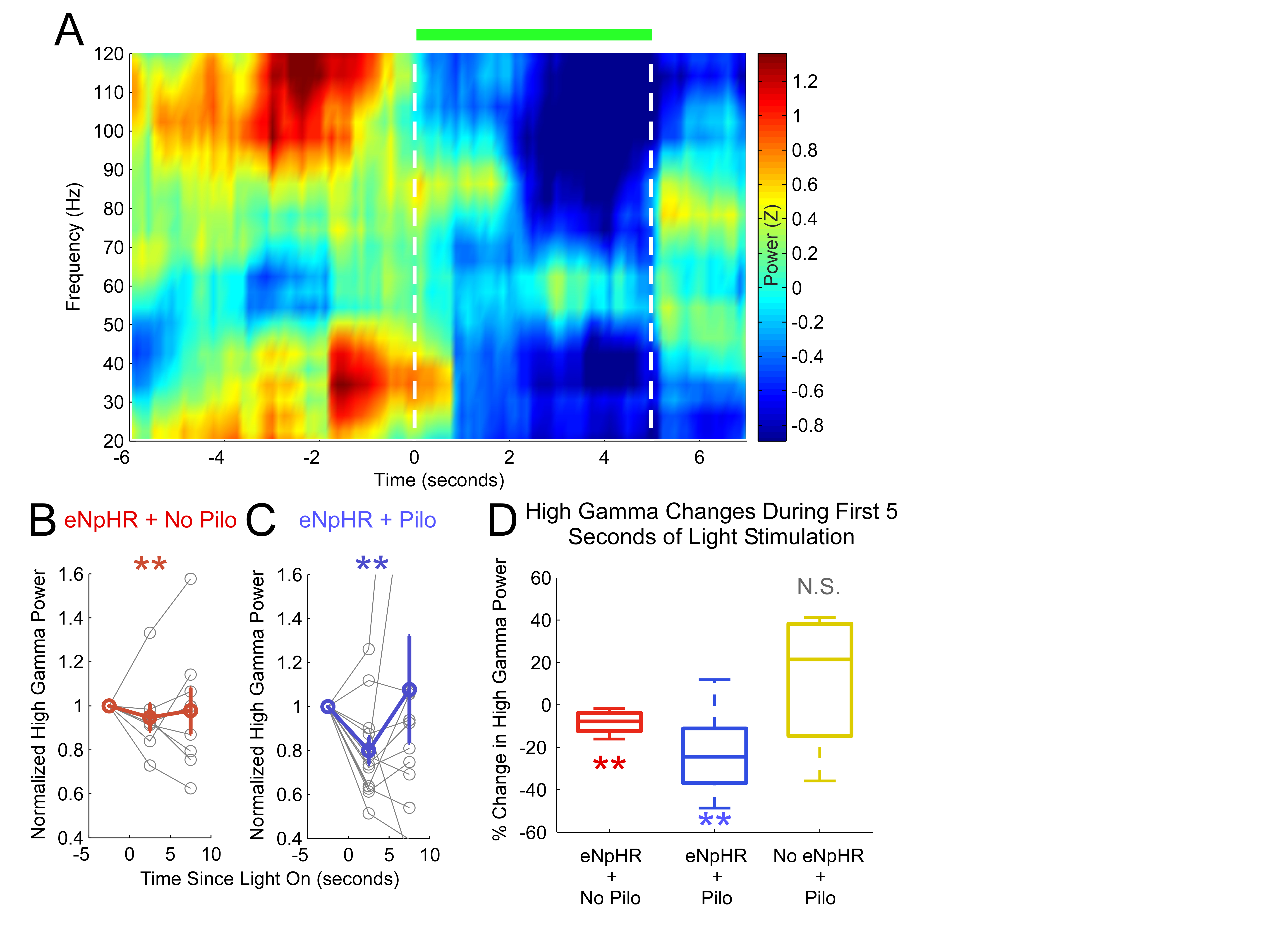

Supplement: Figure S1 — High-gamma power is transiently suppressed during illumination. A. Averaged LFP spectrogram in response to 5 second pulses of light delivered with 30 second intervals between each pulse (N = 35 pulses). Note the clear decrease in high-gamma power across most frequencies during the periods of illumination (green bar). B, C. High-gamma power is decreased during the first 5 seconds of prolonged illumination (lasting 1–3 minutes) in animals expressing eNpHR, but not during any subsequent 5 second periods. This is true both in the absence (B) and presence (C) of pilocarpine to induce seizures. D. Bar and whisker plot of changes in high-gamma power during the first 5 seconds of illumination. Power is decreased in animals expressing eNpHR (red and blue bars), but is non-significantly increased in control animals that do not express eNpHR. This suggests that light-activated hyperpolarization of eNpHR expressing pyramidal cells is playing a role in decreasing high-gamma power. Median (horizontal line), 25–75 percentiles (box) and 2.5–97.5 percentile of the data (whiskers) are shown. (TIFF) [file pone.0062013.s001.tiff]
