# Supplementary material for: Optogenetic Delay of Status Epilepticus Onset in an In Vivo Rodent Epilepsy Model
Source: PLoS One. 2013 Apr 24;8(4):e62013. doi: 10.1371/journal.pone.0062013 (PMC3634849; doi:10.1371/journal.pone.0062013)
Supplement: Methods S1 — Histological processing of the brain and immunohistochemistry. The brains were dissected and processed for histology for injection site/electrode location and virus expression confirmation. Following postfixation in 10% formalin for 2–4 hours at room temperature, brains were cryoprotected in PBS containing 20% sucrose and 0.02% sodium azide at 4°C, cut in serial coronal sections 40 µm thick on a freezing microtome and stored in PBS-azide. Sections were mounted on slides and evaluated for direct identification of YFP expressing cells using fluorescent microscope. For histological evaluation sections were stained with Cresyl Violet or Hematoxylin and Eosin. Fluorescence immunostaining of free-floating sections was performed to visualize endogenous expression of activity-regulated immediate early gene Arc and to amplify YFP signal from the viral transgene. Briefly, sections were blocked for 1 hour at room temperature with 10% (v/v) normal goat serum (NGS, Jackson Immunoresearch) in PBS containing 0.1% (v/v) Triton X-100 (PBST) and then incubated overnight at 4°C with primary antibodies diluted in PBST containing 3% NGS. A monoclonal mouse anti-GFP antibody that recognizes YFP (clone 3E6, Invitrogen) was used at 1∶1000 dilution, and a polyclonal rabbit anti-Arc antibody (gift of Dr. P. Worley) was used at 1∶4000 dilution. After four rinses with PBST, sections were incubated for 2 hours at room temperature with secondary antibodies, Alexa 488-conjugated goat anti-mouse IgG and Alexa 633-conjugated goat anti-rabbit IgG (Invitrogen), each at 1∶250 dilution. Sections were rinsed four times with PBST and mounted on slides with Fluoromount (Sigma-Aldrich). Sections were evaluated and images acquired using an inverted epifluorescence microscope (Nikon) equipped with appropriate filters to selectively visualize each fluorophore, and montages covering the hippocampus were assembled using Volocity software (PerkinElmer). (DOCX) [file pone.0062013.s002.docx]

**Supporting Information Legends**

**Methods S1**

**Histological processing of the brain and immunohistochemistry**

The brains were dissected and processed for histology for injection site/electrode location and virus expression confirmation. Following postfixation in 10% formalin for 2-4 hours at room temperature, brains were cryoprotected in PBS containing 20% sucrose and 0.02% sodium azide at 4^º^ C, cut in serial coronal sections 40 μm thick on a freezing microtome and stored in PBS-azide. Sections were mounted on slides and evaluated for direct identification of YFP expressing cells using fluorescent microscope. For histological evaluation sections were stained with Cresyl Violet or Hematoxylin and Eosin.

Fluorescence immunostaining of free-floating sections was performed to visualize endogenous expression of activity-regulated immediate early gene Arc and to amplify YFP signal from the viral transgene. Briefly, sections were blocked for 1 hour at room temperature with 10% (v/v) normal goat serum (NGS, Jackson Immunoresearch) in PBS containing 0.1% (v/v) Triton X-100 (PBST) and then incubated overnight at 4ºC with primary antibodies diluted in PBST containing 3% NGS. A monoclonal mouse anti-GFP antibody that recognizes YFP (clone 3E6, Invitrogen) was used at 1:1000 dilution, and a polyclonal rabbit anti-Arc antibody (gift of Dr. P. Worley) was used at 1:4000 dilution. After four rinses with PBST, sections were incubated for 2 hours at room temperature with secondary antibodies, Alexa 488-conjugated goat anti-mouse IgG and Alexa 633-conjugated goat anti-rabbit IgG (Invitrogen), each at 1:250 dilution. Sections were rinsed four times with PBST and mounted on slides with Fluoromount (Sigma-Aldrich). Sections were evaluated and images acquired using an inverted epifluorescence microscope (Nikon) equipped with appropriate filters to selectively visualize each fluorophore, and montages covering the hippocampus were assembled using Volocity software (PerkinElmer).
